# Full sib pens of pigs are not suitable to identify variance component of associative effect: a simulation study using Gibbs Sampling

**DOI:** 10.1186/1471-2156-10-9

**Published:** 2009-02-27

**Authors:** Jiqiu Cheng, Steven Janssens, Nadine Buys

**Affiliations:** 1 Livestock genetics, Department Biosystems, K.U.Leuven, Kasteelpark Arenberg 30 – bus 2456, 3001 Heverlee, Belgium

## Abstract

**Background:**

Accounting for and quantifying the associative effect of each animal could improve both welfare of animals and response to selection. Because of the limitation of REML, Gibbs Sampling could be an alternative technique to estimate the variance component of the associative effect. The objective of this study was to investigate the estimation accuracy of the variance component of associative effect by using simulation via Gibbs Sampling. The simulated data comprised five generations of pigs. The breeding animals of each generation were selected randomly. In the simulation, variations were introduced for the methods of assigning pens (random, mixed sib and full sib), the number of pigs per pen (5 or 10), the number of breeding animals per generation (162 or 324) and the correlation between genetic direct effect and genetic associative effect (-0.5, 0.1 or +0.5). Each set of simulation was run for 30 replications.

**Results:**

Random assignment or mixed sib assignment resulted in bias of estimated variance components in only 3 of 24 combinations. Furthermore, these 3 cases occurred with 162 breeding animals. With full sib assignment, 9 out of 12 groups of estimates significantly deviated from the true parameter value. The Root Mean Square Errors obtained with the full sib assignment were higher than with the other two methods of pen assignment in most of the cases. The Root Mean Square Errors obtained with datasets with 324 breeding animals were notably smaller than the datasets from 162 breeding animals. Within each method of pen assignment, the relative bias of the associative effect was significantly smaller with group size 10 than with group size 5.

**Conclusion:**

Full sib assignment caused difficulties to estimate variance components in most of the cases, due to a lack of identifiability. With random and mixed assignment, most data structures yielded unbiased results but increasing the number of breeding animals or group size improves the estimation. Thus to get identifiable and unbiased estimates of the genetic associative effect, it is recommended to avoid close genetic relationship between animals within one pen and to use sufficient numbers of breeding animals and sufficient group sizes.

## Background

Social interactions are commonly observed among group housed livestock and some of them have negative effects, such as: aggressive behaviour for social rank and competition for limited resources (food, water, space). These interactions may become more important when resources are more restricted leading to increase of injuries and decrease of productivity [1,2]. Artificial selection ignoring these interactions has been proven to result in a suboptimal selection response [3,4]. Thus quantifying and accounting for the interaction among animals could improve both the welfare of animals and the response to selection.

According to Griffing [3], the interaction among animals is defined as a competitive effect. The genetic direct effect refers to the effect of the genes of the individual itself and is expressed in its own phenotype. Instead, the genetic associative effect of an animal refers to genes that influence the performance of its penmates. Attempts have been made to estimate both the genetic direct effect and the genetic associative effect using performance and pedigree data (breeding value estimation). Estimation of the magnitude of the associative effect has been performed employing Restricted Maximum Likelihood (REML) [5-7]. However, the REML estimator relies on an asymptotic distribution, which means the inferences are valid strictly for a sample of infinite size [8-10]. Therefore it is difficult to calculate reliable confidence intervals around REML variance components parameters.

Implementation of Bayesian method may provide a valuable alternative. Bayesian Markov Chain Monte Carlo (MCMC) methods have been introduced to quantitative genetics since the early 1990s [9]. Gibbs Sampling has been frequently applied to animal breeding and plant breeding [11]. However, until now few studies have been undertaken to investigate the accuracy of the estimation of the associative effect via Gibbs Sampling [8]. Moreover, the impact of the data structure affected by methods to assign pigs to pens, group size (pigs/pen) or the population size simultaneously on the estimation of variance components has not yet been studied.

The objective of this study was to investigate the accuracy of the posterior means of variance components in relation to the (genetic) relationships among penmates, the number of pigs per pen and the variation of the number of breeding animals.

## Results

### Simulation results

In this study, datasets were simulated for 36 combinations of parameters. Each set of parameters was replicated 30 times. Simulations were programmed in R 2.5.1 [12] and performed on the cluster computer 'VIC' at the K. U. Leuven.

### Posterior distribution

Initial tests indicated that a total length of the Gibbs chain of 550000 was sufficient. The burn-in period was fixed at 50000, which means that the first 50000 samples were discarded. To reduce the correlation between Gibbs samples, a conservative thinning rate of 500 was used. Therefore, 1000 Gibbs samples of each chain were saved. Geweke and Gelman diagnostics were used to access the convergence of Gibbs Samples. With respect to full sib assignment, less than 33% of the replications gave a diagnostic value around 1.2, whereas 95% of the replications with the random and mixed sib assignment had a diagnostic value around 1.02.

**Figure 1:**
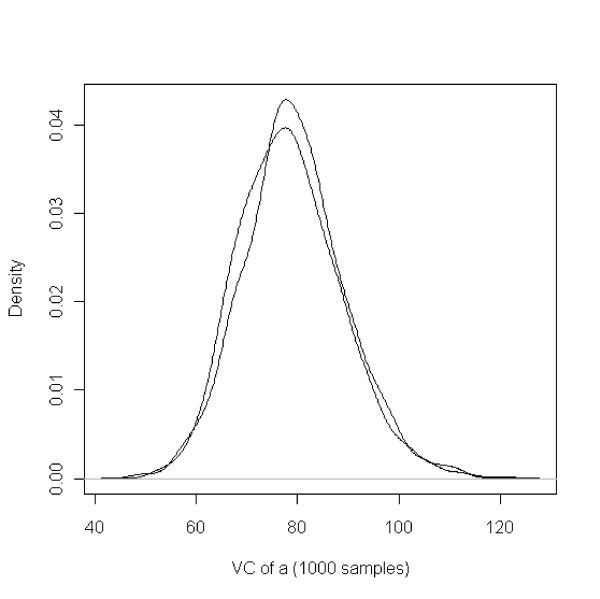
**Density plots of variance component of genetic associative effect from 2 Gibbs sampling chains**. Each chain contains 1000 Gibbs samples after the burn-in period.

The density plots of Gibbs samples of the variance of the genetic associative effect from two chains were shown in figure 1. The overlap observed in the density plot indicates convergence of the Gibbs sampling process. The mean of the Gibbs Samples was used as the estimated value for each replication. Thus, for each combination of parameters, we obtained 30 estimates for each variance component.

### Confirmation of theoretical justification

Estimation with a model considering only genetic direct effect (and *e*) yielded ${\hat{\sigma }}_{d}^{2}$ = 1699.40, ${\hat{\sigma }}_{e}^{2}$ = 3830.93. Compared with the true parameter set: $\sigma_{d}^{2}$ = 1250, *σ*_*ad *_= 140, $\sigma_{a}^{2}$ = 62.5 and $\sigma_{e}^{2}$ = 3687, the estimations of both $\sigma_{d}^{2}$ and $\sigma_{e}^{2}$ were inflated. It confirms that ignoring the genetic associative effect results in bias of estimations of the other variance components.

### Parameter estimates

The mean and the standard error of estimated values (*d, a, cov *and *e*) from 30 replications were presented in table 1. With full sib assignment we observed only 1 out of 12 groups where all estimates did not significantly deviate from the true parameter values. The only exception where full sib assignment yielded unbiased results was with S_324 _(324 breeding animals per generation), a correlation of 0.5 and group size of 5. Moreover, most of the standard errors calculated from the combinations with full sib assignment were high compared to the other two methods of assignment.

**Table 1:** The mean and standard error of posterior distribution of *d*, *a *and *e*

**Correlation** **(d & a)**	**Number of breeding animals per Generation**	**Pen Assignment**	**Pen size**	${\hat{\sigma }}_{d}^{2}$	${\hat{\sigma }}_{da}$	${\hat{\sigma }}_{a}^{2}$	${\hat{\sigma }}_{e}^{2}$
**-0.5** **(*σ*_*da *_= -140)**	**162^a^**	**Random**	**5**	1257 (36.66)	-133 (6.69)	58 (2.43)	3721 (25.53)
			**10**	1239 (29.59)	-133 (5.19)	62 (1.46)	3699 (21.69)
		**Mixed sib**	**5**	1250 (35.65)	-128 (6.64)	56* (2.27)	3706 (25.99)
			**10**	1261 (31.17)	-139 (5.56)	62 (1.33)	3689 (25.74)
		**Full sib**	**5**	850** (50.72)	-60** (9.35)	43**(2.37)	3944**(30.92)
			**10**	710**(58.63)	-70**(9.65)	51**(1.96)	4031**(41.47)
		
	**324^b^**	**Random**	**5**	1245 (19.78)	-133 (3.08)	60 (1.68)	3715 (15.80)
			**10**	1248 (16.14)	-139 (3.76)	63 (1.05)	3698 (15.85)
		**Mixed sib**	**5**	1235 (20.77)	-143 (4.55)	63 (1.85)	3694 (16.80)
			**10**	1228 (21.44)	-141 (3.45)	63 (0.94)	3703 (18.28)
		**Full sib**	**5**	1054** (62.21)	-92**(11.74)	46**(3.35)	3842**(42.21)
			**10**	933** (88.04)	-102**(12.23)	57*(2.17)	3896**(54.36)

**0.1** **(*σ*_*da *_= 27.95)**	**162^a^**	**Random**	**5**	1224 (24.39)	34 (4.54)	58* (1.99)	3738* (19.61)
			**10**	1256 (28.39)	27 (5.68)	61 (1.69)	3694 (20.39)
		**Mixed sib**	**5**	1239 (27.44)	29 (4.15)	60 (2.17)	3713 (21.97)
			**10**	1166* (31.58)	28 (4.10)	62 (1.68)	3763**(20.99)
		**Full sib**	**5**	996** (55.67)	69** (4.94)	56** (2.88)	3833** (26.80)
			**10**	917** (49.94)	72** (5.43)	56** (1.25)	3889** (29.92)
		
	**324^b^**	**Random**	**5**	1277 (23.43)	32 (4.02)	61 (1.90)	3688 (13.70)
			**10**	1223 (19.12)	24 (3.51)	63 (1.17)	3712 (13.76)
		**Mixed sib**	**5**	1268 (24.29)	30 (3.88)	58 (1.73)	3697 (18.06)
			**10**	1253 (22.33)	32 (3.42)	62 (1.01)	3694 (17.85)
		**Full sib**	**5**	1170 (55.26)	62** (7.67)	51** (3.33)	3767** (29.39)
			**10**	940** (54.06)	65** (8.20)	57** (1.68)	3891** (36.79)

**0.5** **(*σ*_*da *_= 140)**	**162^a^**	**Random**	**5**	1266 (27.38)	146 (4.88)	63 (2.17)	3689 (18.15)
			**10**	1256 (32.15)	144 (4.03)	64 (1.68)	3685 (21.26)
		**Mixed sib**	**5**	1204 (33.05)	134 (4.42)	60 (2.33)	3732 (25.91)
			**10**	1209 (25.53)	138 (4.64)	62 (1.23)	3728 (21.33)
		**Full sib**	**5**	1256 (60.26)	133 (5.33)	72* (3.58)	3653 (30.03)
			**10**	1137** (42.95)	141** (4.71)	66** (1.81)	3748** (25.16)
		
	**324^b^**	**Random**	**5**	1269 (17.75)	140 (3.46)	62 (1.68)	3693 (12.83)
			**10**	1229 (20.36)	142 (2.94)	62 (1.18)	3718 (16.22)
		**Mixed sib**	**5**	1224 (20.01)	139 (3.44)	63 (1.34)	3709 (11.61)
			**10**	1247 (21.94)	136 (3.13)	63 (0.76)	3701 (19.26)
		**Full sib**	**5**	1259 (34.83)	142 (3.15)	63 (2.25)	3668 (21.39)
			**10**	1156* (37.24)	141 (3.84)	64 (0.91)	3730 (21.42)

*:p-value < 0.05, **:p-value < 0.01.

*d*: genetic direct effect; *a*: genetic associative effect; *e*: random residual.

a: 162 breeding animals represent 150 sows and 12 boars.

b: 324 breeding animals represent 300 sows and 24 boars.

Values used in the simulation: $\sigma_{d}^{2}$ = 1250, $\sigma_{a}^{2}$ = 62.5, $\sigma_{e}^{2}$ = 3687, *σ*_*da *_= -140, 27.95 or 140.

With S_324_, estimates obtained from random pen assignment or mixed sib pen assignment with different group sizes and correlations were not significantly different from the true parameter values. When there were 162 breeding animals per generation, 3 estimates were significantly different from the true parameter values at the 5% significance level (p-value < 0.05). This occurred with the following combinations: random pen assignment, correlation 0.1 and group size 5; mixed sib assignment, correlation 0.1 and group size 10; mixed sib assignment, correlation -0.5 and group size 5.

### Root Mean Square Error (RMSE)

**Figure 2:**
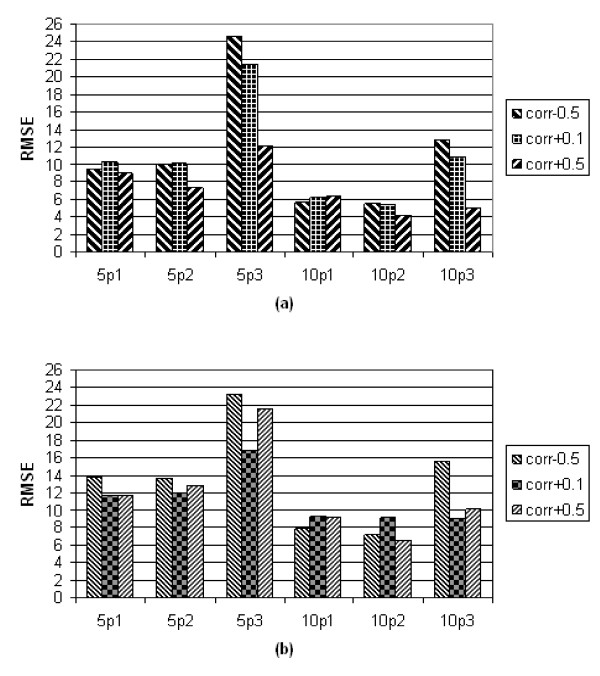
**Plot of RMSE with (a) 324 breeding animals and (b) 162 breeding animals**. Left nine columns represent group size 5; right nine columns represent group size 10; corr: correlation; p1: random assignment; p2: mixed sib assignment; p3: full sib assignment; Y axis represents the root mean square error of the estimated genetic associative variance.

The RMSE of the variance of associative effect obtained from 36 parameter sets were shown in figure 2(a) and 2(b). The RMSE was smaller with S_324 _compared to the cases with S_162_, but not significantly different (p-value = 0.06). Within the same number of breeding animals, the RMSE obtained from group size 5 were significantly higher than the cases from group size 10 (S_162: _p-value = 0.03; S_324: _p-value = 0.005). Within the same level of group size, the RMSE of random sib assignment (p1) are not significantly different from the mixed sib assignment (p2) (group size 5: p-value = 1; group size 10: p-value = 0.26).

With the method of full sib assignment (p3) and the following combinations, datasets yielded higher RMSE compared to assignment methods p1 and p2: S_324_, correlation -0.5; S_324_, correlation +0.1; S_162_, correlation +0.1; S_162_, correlation +0.5. The RMSE from the full sib assignment depended on correlations, indicating that there may be an association between full sib assignment and "correlation" (between *d *&*a*) on the uncertainty of estimates for the associative effect.

### Percent relative bias

The results from MANOVA test of relative bias showed a significant interaction between the number of breeding animals, the method of pen assignment and the correlation (Wilk's Lambda test: F value = 2.21, p-value = 0.0037). Interaction between pen assignment method and group size (Wilk's Lambda test: F value = 9.04, p-value < 0.0001) was also significant at the family wise significance level of 0.1 (the significance level for the individual test is 0.006).

**Figure 3:**
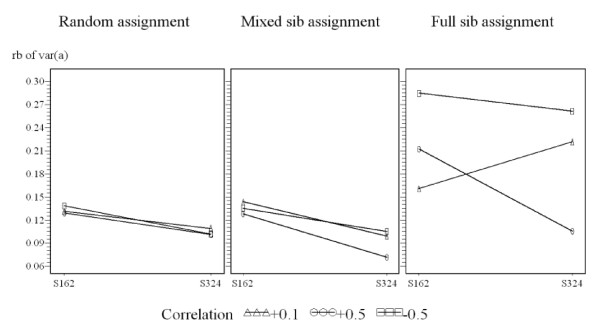
**Plot of relative bias of genetic associative variance applying three assignment methods**. Three assignment methods: random assignment, mixed sib assignment, full sib assignment; a: genetic associative effect; S162: 162 breeding animals per generation, S324: 324 breeding animals per generation; X axis represents the number of breeding animals; Y axis represents the relative bias of the estimated genetic associative variance.

With random assignment, the relative bias of the associative effect was not significantly different between three correlations and two numbers of breeding animals (figure 3). For the mixed sib assignment with correlation +0.5, the relative bias of the associative effect for S_324 _was significantly smaller than the case for S_162 _(difference = 0.06, p-value = 0.005) (figure 3).

The plot for full sib assignment (figure 3) illustrated that there was no significant difference between S_162 _and S_324 _when the correlation equalled -0.5 (p-value = 0.2531) (figure 3). However, the difference became significant when the true correlation equalled 0.1 or +0.5 (correlation = +0.1, p-value = 0.0014; correlation = +0.5, p-value < 0.0001). When the true correlation was +0.5, the relative bias from S_324 _was significantly smaller, and when the true correlation was +0.1, the relative bias from S_324 _was significantly larger than from S_162_.

**Figure 4:**
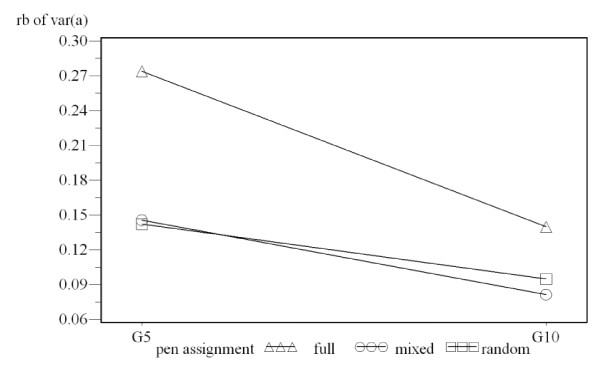
**Plot of relative bias of genetic associative variance with two group sizes**. G5: group size 5; G10: group size 10; random: random assignment, mixed: mixed sib assignment, full: full sib assignment; X axis represents the number of animals per pen; Y axis represents the relative bias of the estimated genetic associative variance.

The relative bias of the variance of associative effect obtained from group size 10 was smaller than from group size 5 for each pen assignment method (figure 4). The difference was confirmed by contrast tests (random assignment: -0.047, p-value < 0.0001; mixed full sib assignment: -0.064, p-value < 0.001; full sib assignment: -0.133, p-value < 0.001). With group size 10 or 5, the difference of relative bias between random assignment and mixed full sib assignment was not significant. With group size 10, relative bias of the associative effect obtained from full sib assignment was 0.045 higher than from random assignment (p-value < 0.0001) and 0.132 higher than from random assignment with group 5 (p-value < 0.0001).

## Discussion

In this study, we simulated average daily gain of pigs for 36 data structures combining 4 parameters; the number of breeding animals per generation, correlation between *d *&*a*, methods of pen assignment and group size. The simulated pedigree structure of 5 generations represented a realistic breeding scheme.

Our results showed that ignoring the genetic associative effect led to overestimations of $\sigma_{d}^{2}$ and $\sigma_{e}^{2}$, which was in agreement with Cappa and Cantet [13]. In our study, *σ*_*ad *_was set to +140 in the simulation. As mentioned in the method part, the biases of $\sigma_{d}^{2}$ and $\sigma_{e}^{2}$ consist of the sum of two terms: $\left(\sum_{j=1}^{4}A_{AQ_{j}}+\sum_{i=1}^{4}A_{BP_{i}}\right)\sigma_{ad}$ and $\left(\sum_{i=1}^{4}\sum_{j=1}^{4}A_{P_{i}Q_{j}}\right)\sigma_{a}^{2}$. Thus these two terms and the sum of them were all positive, which inflated the estimations of $\sigma_{d}^{2}$ and $\sigma_{e}^{2}$.

Cappa and Cantet performed a simulation of genetic associative effect among forest trees using Gibbs Sampling [13]. In their study, simulation parameters were ${\hat{\sigma }}_{d}^{2}$ = 12.553, ${\hat{\sigma }}_{ad}$ = -3.126, ${\hat{\sigma }}_{a}^{2}$ = 1.259 and ${\hat{\sigma }}_{e}$ = 5.189. When the genetic associative effect was excluded from the model, ${\hat{\sigma }}_{d}^{2}$ was 10.644 and ${\hat{\sigma }}_{e}^{2}$ was 9.257, i.e., was overestimated. The reason was that *σ*_*ad *_used in their study had a negative value. Thus the biases of $\sigma_{d}^{2}$ and $\sigma_{e}^{2}$ depended on the magnitude of $\left(\sum_{j=1}^{4}A_{AQ_{j}}+\sum_{i=1}^{4}A_{BP_{i}}\right)\sigma_{ad}$ and $\left(\sum_{i=1}^{4}\sum_{j=1}^{4}A_{P_{i}Q_{j}}\right)\sigma_{a}^{2}$. When the magnitude of the second term was larger than the first term, the biases were positively inflated.

With full sib assignment, 11 out of 12 combinations showed biased estimations at the 1% significance level. Random assignment or mixed sib assignment resulted in bias of estimated variance components in only 3 of 24 combinations. Moreover, all these three biased results were found with 162 breeding animals only. Thus random composition and mixed sib composition of pen groups resulted in unbiased estimates of variance components. The more penmates are genetically related, the more difficult it is to separate variance components. Estimation accuracy benefits from larger numbers of breeding animals.

Cantet and Cappa have pointed out recently that models with genetic direct effect and genetic associative effect may face the problem of identifiability [14]. Identifiability means that parameters are weakly identified because the data provide little information [15]. Bayesian non-identifiability is equivalent to lack of identifiability in likelihood [15]. Cantet and Cappa have shown that the variance components are identifiable only and only if the smallest eigenvalue of the restricted maximum likelihood (REML) information matrix is positive.

To investigate the identifiability of the cases which resulted in biased estimations, we selected several pairs of pens from the simulated data randomly with full sib assignment and calculated the eigenvalues of information matrix using the method introduced by Cantet and Cappa. The genetic relationship coefficients between pigs in different pens ranged from 0.02 to 0.07. The results indicated that two eigenvalues of the information matrix were zero, i.e. the information matrix is singular and parameters can not be estimated separately. Thus we suspect that the biased estimations of the parameters with full sib assignments are due to the lack of the identifiability.

In Cantet and Cappa's study, the problem of identifiability was related to the confounding between the genetic associative effect and fixed pen effect. In our study, we did not include a variance component for pens in the simulation model. It implied that not only the fixed pen effect, but also the assignment methods play a role in identifying variance components separately. Cantet and Cappa have illustrated this in a theoretical way. We confirmed this by the simulation. In addition, Cantet and Cappa suggested an alternative model with different intensities of competition for each animal by exchanging animals between pens time by time. Although it could improve the identifiability theoretically, it is difficult to implement this in reality.

Gelfand and Sahu mentioned that a consequence of a non-identifiable model is the possibility of drifting which could result in the difficulty of convergence. In our study the Gelman diagnostics computed on Gibbs chains with full sib assignment were slightly above 1 for about one third of the replicates. This may indicate the poor convergence in some replicates. Both the biased estimates and a poor quality of convergence may be due to the identifiability issue.

Our results obtained by Gibbs Sampling confirmed the results of the study of Bijma and Muir's using ASREML [16]. They also investigated the data structure used by Wolf [17] and found that the variance of genetic associative effect was not identifiable when group members had a strong genetic relationship. They suggested using random composition of pens. We also found that the variance components obtained with the full sib assignment were biased. In addition, not only random assignment but also mixed sib assignment provided unbiased estimations of variance components in most cases.

Within each method of pen assignment, the relative bias of the variance of associative effect was significantly smaller with group 10 than group size 5. It indicates that estimations with larger group size produce smaller relative bias than smaller group size.

With respect to the uncertainty of estimations, most RMSE obtained with the method of full sib assignment were considerably larger than cases with the other two methods of pen assignments, which was inconsistent with the results of Van Vleck [18]. Van Vleck simulated data with similar pen assignment methods: full sib assignment, random assignment and mixed full sib assignment (half of the penmates are full sibs from one litter and the other half from another litter). He found that there was no problem to separate variance components for all these three methods of pen assignments and the standard deviation obtained from the full sib assignment was the smallest among the three pen assignments. In our results, it was hard to separate *d & a *and the standard deviation of *a *was large in the most cases. However, the estimation was unbiased with the combination: correlation +0.5, S_324 _and full sib assignment. Furthermore, the RMSE obtained from that combination was also similar to the other combinations with full sib assignment. Van Vleck [18] only looked at the influence of assignment methods without varying any of the other parameters that we simulated. We also found one combination of parameters that gave unbiased results. But the other 11 combinations showed problems obviously. It is concluded that in general full sib assignment should be avoided to generate data from which an associative effect is to be estimated.

## Conclusion

It is concluded that ignoring the genetic associative effect causes biased variance components which could lead to unrealistic selection perspective. The estimation of the genetic associative effect via Gibbs Sampling is dependent on the data structure. Investigation of the required minimum number of breeding animals per generation and the number of penmates per pen is therefore warranted before starting real experiments. Furthermore, full sib assignment should be avoided as an experimental design to determine associative effect.

## Methods

### Simulation model

Datasets were simulated for a pure bred population and a single trait (average daily gain of pigs, g/day). The phenotype of individuals was composed of heritable and non-heritable effects. We assumed the associative effect to be (partially) genetic and expressed in the performance of the penmates. The simulation model included one fixed effect (sex) and three random effects: genetic direct effect, genetic associative effect and random error [16].

*Y *= *Xb *+ *Z*_*d*_*d *+ *Z*_*a*_*a *+ *e*

Y represented the average daily gain of the pig; *b *indicated the effect of sex on the average daily gain (+20 for males and -20 for females); *d *represented the genetic direct effect; *a *represented the genetic associative effect, affecting the growth of penmates; *e *represented the independent random residual of the individual pig; X, *Z*_*d*_, *Z*_*a *_were incidence matrix of each effect corresponding to each individual.

It was assumed that the random effects follow a multivariate normal distribution:

$$\begin{array}{ccc} \left(\begin{array}{c} d\\ a\\ e \end{array}\right)∼N(0,\sum ), & \sum =\left(\begin{array}{cc} G & 0\\ 0 & I\sigma_{e}^{2} \end{array}\right), & G=\left(\begin{array}{cc} A\sigma_{d}^{2} & A\sigma_{da}\\ A\sigma_{da} & A\sigma_{a}^{2} \end{array}\right) \end{array}$$

$\sigma_{d}^{2}$, $\sigma_{a}^{2}$ were the variances of genetic direct effect and genetic associative effect respectively; *σ*_*da *_was the covariance between *d *&*a*; $\sigma_{e}^{2}$ was the variance of the random residuals, accounting for the non-genetic effect. It was assumed that the residuals of each pig are independent. A represented the numerator relationship matrix, which accounted for the pedigree information of animals. Σ was the variance-covariance matrix of random effects *d, a *and *e*. G was the variance-covariance matrix of genetic effects *d *and *a*.

For an individual pig *i*, the average daily gain was simulated as

$$y_{i}=b_{i}+d_{i}+\sum_{j=1}^{p}a_{ji}+e_{i}$$

*d*_*i *_denoted the genetic direct effect of the individual *i*; *a*_*ji *_denoted the genetic associative effect coming from the *j*^*th *^penmate of the *i*^*th *^individual; *p *denoted the number of penmates of the *i*^*th *^individual; *e*_*i *_denoted the random residual of the *i*^*th *^individual.

Based on the Cholesky decomposition [19], G was separated into a product of a matrix and its transpose. For the animals from the base generation, *d *and *a *were simulated using the partitioned matrix obtained from G multiplied by standard random normal deviates. For the following generations (progeny), *d *and *a *were the sum of the average of the *d *and *a *of the parents and a random Mendelian sampling term. *e *was simulated as the deviation of random residuals multiplied by a standard random normal deviate.

### Simulation structure

Simulation included five generations of pigs, and breeding animals for each generation were selected randomly. Litter size followed a normal distribution varying from 2 to 20 with mean equal to 9 and standard deviation equal to 2.6. Sex effect was simulated assuming a uniform distribution, i.e. the probability of male or female was 50%. Since litter size was not fixed, the total number of animals in each replication was not fixed.

In order to investigate the impact of the data structure on the estimation of variance components, a number of data sets were simulated. The influence of pen composition (assignment of pigs to pens), group size (pigs/pen), number of breeding animals and the covariance (cov) between *d & a *were examined. Three different scenarios were used to assign pigs to pens. The first scenario was to assign pigs randomly (p1). The second one was to assign half of the pens with full sibs and the other half non full sibs (p2) and the third one was to assign full sibs per pen (p3) [6,20]. Within each pen assignment scenario, 162 and 324 breeding animals (S_162 _and S_324_), two different group sizes (5 and 10) and three different correlations between d & a (-0.5, +0.1, +0.5) were specified.

### Statistical methods

Variance components were analysed using the Multiple Trait Gibbs Sampler for Animal Models (MTGSAM) [11] and applying the same statistical model as the one used for simulation. In its original version, MTGSAM could only handle models with direct and maternal genetic effects. For this study we modified the program and extended it to include the genetic associative effect.

Two Gibbs chains using different sets of starting values were run to check the convergence. The Geweke and Gelman diagnostic [21,22] were applied to determine the convergence of two Markov chains. For further analysis, results were summarized over 30 replicates (mean and standard errors were calculated from the 30 groups of posterior means of variance components). Both the Wilcoxon rank sum test and Student t-test were used to test the deviation of the mean from the real parameter values. Root Mean Square Error (RMSE) was used to measure the uncertainty of the estimated values. The RMSE of the estimator $\hat{\theta }$ was calculated as [23]:

$$\text{RSME }(\hat{\theta })=\sqrt{E\left({\left(\hat{\theta }-\theta \right)}^{2}\right)}=\sqrt{\frac{\sum_{i=1}^{n}{\left({\hat{\theta }}_{i}-\theta \right)}^{2}}{n}}$$

where $\hat{\theta }$ is the estimated value of parameters obtained in each replication; *θ *is the true value used for simulation and n is the number of replications.

The accuracy of the parameter estimates was quantified by the percent relative bias. The percent relative bias was calculated as $\frac{\hat{\theta }-\theta }{\theta }$[24]. To study the effects of pen assignment, group size, number of breeding animals and the correlation between *d *&*a *simultaneously on the percent relative bias of the four variance components (d, cov, a and e), multivariate analysis of variance (MANOVA) was used (GLM, SAS 9.1.3) [25].

### Theoretical justification for including genetic associative effect

Ignoring the genetic associative effect (if present in the data) will often result in biases of variances of genetic direct effect ($\sigma_{d}^{2}$) and random residual ($\sigma_{e}^{2}$) [13]. Looking into the genetic covariance between animals may help to explain the bias. Suppose animal A has penmates *P*_1_,...,*P*_4_, animal B has penmates *Q*_1_,...,*Q*_4_, and A and B are from two different pens. Based on the statistical model that we used the genetic covariance between A and B was:

$$\begin{array}{l} \mathrm{cov}(A,B)\\ =\mathrm{cov}\left(d_{A}+\sum_{i=1}^{4}a_{P_{i}},\;d_{B}+\sum_{j=1}^{4}a_{Q_{j}}\right)\\ =\mathrm{cov}\left(d_{A},d_{B}\right)+\mathrm{cov}\left(d_{A},\sum_{j=1}^{4}a_{Q_{j}}\right)+\mathrm{cov}\left(d_{B},\sum_{i=1}^{4}a_{P_{i}}\right)+\mathrm{cov}\left(\sum_{i=1}^{4}a_{P_{i}},\sum_{j=1}^{4}a_{Q_{j}}\right)\\ =A_{AB}\sigma_{d}^{2}+\left(\sum_{j=1}^{4}A_{AQ_{j}}+\sum_{i=1}^{4}A_{BP_{i}}\right)\sigma_{ad}+\left(\sum_{i=1}^{4}\sum_{j=1}^{4}A_{P_{i}Q_{j}}\right)\sigma_{a}^{2} \end{array}$$

If we consider A and B to be half sibs, then the genetic relationship between A and B is 0.25, i.e. *A*_*AB *_= 0.25. To make sure that the covariance between A and B is an unbiased estimation of $\sigma_{d}^{2}$, $\left(\sum_{j=1}^{4}A_{AQ_{j}}+\sum_{i=1}^{4}A_{BP_{i}}\right)\sigma_{ad}+\left(\sum_{i=1}^{4}\sum_{j=1}^{4}A_{P_{i}Q_{j}}\right)\sigma_{a}^{2}$ should be zero. If *σ*_*ad *_and $\sigma_{a}^{2}$ are not equal to zero, the coefficients related to *σ*_*ad *_and $\sigma_{a}^{2}$ should be zero. That means animal A is unrelated to the penmates of B and animal B is unrelated to the penmates of A. Moreover, there should be no genetic relationships between penmates *P*_1_,...,*P*_4 _and *Q*_1_,...,*Q*_4_. However, it is unrealistic to assume that all animals are genetically unrelated. Thus the terms related to *σ*_*ad *_and $\sigma_{a}^{2}$ will be non-zero and affect the estimation of $\sigma_{d}^{2}$. The magnitude of bias depends on the magnitude of the terms related to *σ*_*ad *_and $\sigma_{a}^{2}$. Only when $\left(\sum_{j=1}^{4}A_{AQ_{j}}+\sum_{i=1}^{4}A_{BP_{i}}\right)$*σ*_*ad *_and $\left(\sum_{i=1}^{4}\sum_{j=1}^{4}A_{P_{i}Q_{j}}\right)$$\sigma_{a}^{2}$ have the same magnitude and opposite sign, the bias is zero.

To investigate the bias of $\sigma_{e}^{2}$, consider animals A and B have no genetic relationship, i.e. *A*_*AB *_= 0. If the genetic associative effect is ignored, the variation caused by *σ*_*ad *_and $\sigma_{a}^{2}$ will flow into the variance of the residual variance $\sigma_{e}^{2}$ leading to bias of $\sigma_{e}^{2}$. In order to reduce the bias introduced by *σ*_*ad *_and $\sigma_{a}^{2}$, one possibility is to have $\left(\sum_{j=1}^{4}A_{AQ_{j}}+\sum_{i=1}^{4}A_{BP_{i}}\right)$ and $\left(\sum_{i=1}^{4}\sum_{j=1}^{4}A_{P_{i}Q_{j}}\right)$ equal to zero, which is unrealistic as mentioned before. Similarly, the bias of estimation of $\sigma_{e}^{2}$ depends on the magnitude of $\left(\sum_{j=1}^{4}A_{AQ_{j}}+\sum_{i=1}^{4}A_{BP_{i}}\right)$*σ*_*ad *_and $\left(\sum_{i=1}^{4}\sum_{j=1}^{4}A_{P_{i}Q_{j}}\right)$$\sigma_{a}^{2}$. It could be zero when the magnitudes of them are equal with opposite sign.

To confirm this, we took the simulation data obtained from the parameter combination of group size 5, correlation 0.5, 324 breeding animals and mixed full sib assignment. The estimation with a model considering only genetic direct effect and random residual was compared to the true value of simulation parameters.

## Authors' contributions

JC carried out the simulation study, and drafted the manuscript. SJ conceived the study, participated in its design, and revised the manuscript. NB participated in conception, coordinated it and revised the manuscript. All authors read and approved the final manuscript.
